# Health Primers

**Published:** 1880-01

**Authors:** 


Health Primers—The Skin and its Troubles. D. Appleton & Co., N. Y. 1879.

This is a very useful primer, and is calculated to give much useful and practical information on a complex subject.

